# Synthesis of Diatomite-Based Mesoporous Wool-Ball-Like Microspheres and Their Application for Toluene Total Oxidation Reaction

**DOI:** 10.3390/nano10020339

**Published:** 2020-02-17

**Authors:** Quoc-Chon Le, Chinh Chien Nguyen, Thi Thanh Nhi Le, Thierry Lefèvre, Minh Tuan Nguyen Dinh, Sung Hyun Hong, Soo Young Kim, Quyet Van Le

**Affiliations:** 1 Natural Sciences Department, Duy Tan University, Danang 550000, Vietnam; lequocchon@gmail.com; 2 Institute of Research and Development, Duy Tan University, Danang 550000, Vietnam; nguyenchinhchien@duytan.edu.vn (C.C.N.); lethithanhnhi.kh@gmail.com (T.T.N.L.); 3 Département de chimie, Proteo, Cerma, CQMF, Université Laval, Québec, QC G1V 0A6, Canada; thierry.lefevre@chm.ulaval.ca; 4 Faculty of Chemical Engineering, University of Science and Technology, The University of Da Nang, 54 Nguyen Luong Bang, Da Nang 550000, Vietnam; 5 Department of Materials Science and Engineering, Korea University, 145 Anam-ro, Seongbuk-gu, Seoul 02841, Korea; qhrdjakstp@naver.com

**Keywords:** diatomite, surfactant-assisted synthesis, hydrothermal treatment, dissolution–recrystallization, wool-ball-like microsphere, catalyst support

## Abstract

Diatomite (DE) has attracted considerable attention owing to its abundance, low cost, and potential for a wide variety of applications. This work reports the development of mesoporous wool-ball-like (WBL) microspheres from natural DE through a simple hydrothermal treatment. We discovered that the presence of cetyltrimethylammonium bromide is a prerequisite for generating monodispersed WBL microspheres. The mechanism for the transformation of pristine DE into mesoporous microspheres through dissolution–recrystallization was clearly investigated. Interestingly, the microspheres exhibited a specific surface area 25–60 times larger than that of the pristine DE. The application of WBL microsphere DE as an effective support for metallic catalysts in the toluene total oxidation reaction was demonstrated.

## 1. Introduction

Diatomite (DE) is a deposit of various diatomaceous algae, whose size ranges from one to a few micrometers [1]. This material is diverse in shape, highly macroporous, and thermally stable, with a low thermal resistance [2]. Silica is the principal component of DE, constituting approximately 80% to 90% of DE by weight [3]. DE is inexpensive and abundant and has been widely used as a filter aid for pest control, an anticaking agent in grain storage, an absorbent in water treatment [4], a component in construction and insulating materials, a mild abrasive (in toothpaste, facial scrubs, and metal polish), and a catalyst support [5]. New applications have recently been considered, such as a template for thermal energy storage [6], as well as drug delivery [7]. Additionally, DE has been used as a precursor to synthesize new functional materials, i.e., DE-based materials (DBMs) [8,9,10].

The use of DE remains limited for some applications because of its low surface area (1–40 m^2^/g) and low mesoporosity (<0.06 cm^3^/g). To extend the applications of DE, particularly in catalyst support and contaminant absorption, it is essential to make DE more porous, e.g., via diatom zeolitization [11] or by designing DE–carbon hybrid materials [12]. A surfactant-assisted hydrothermal method is commonly used to achieve this goal [13,14,15]. The morphology and porosity of the final material can be controlled by varying the type and concentration of surfactant and by controlling the organization of the final assembly. Among the various surfactants, cetyltrimethylammonium bromide (CTAB) has been most widely employed [13,16,17,18].

It is well known that surfactants can exist as micelles, lamellar structures, clusters, or free molecules and that these organizations can be tuned by changing the concentration [19,20,21], chemical composition, and temperature [22,23]. Such structural assemblies of the surfactant determine the morphology and porosity of the end materials [24,25,26]. The hydrothermal treatment of DE generates materials that are more porous than the initial ones [9,10]. Although the effects of the surfactants on the material morphology during hydrothermal treatment have been studied, several aspects remain unclear, e.g., (1) DE transformation into other materials during the hydrothermal process and (2) the effects of the surfactant on this transformation.

In this study, we first used a surfactant-assisted hydrothermal method to synthesize mesoporous wool-ball-like (WBL) microspheres with a large specific surface area from DE. Then, we investigated DE transformation into WBL microspheres in the presence of NaOH and surfactants. Finally, the microspheres were tested as a support for metallic catalysts in the total oxidation reaction of toluene, which is a volatile organic compound that is dangerous to human health. 

## 2. Experimental Details

### 2.1. Materials

DE fine powder was purchased on the market in Vietnam (VMCGroup, Da Nang, Viet Nam). Sodium hydroxide (NaOH) powder, silver nitrate (AgNO_3_), sodium borohydride (NaBH_4_), manganese (II) nitrate hexahydrate (Mn(NO_3_)_2_·6H_2_O), sodium dodecylbenzenesulfonate (SDBS), and CTAB were purchased from Sigma Aldrich (St. Louis, MO, USA) and were analytical-grade.

### 2.2. Methods

#### 2.2.1. Purification of Diatomite 

Commercial DE may contain inorganic impurities. Thus, the DE was purified using Goren’s method [27]. In a typical procedure, 20 g of original DE powder was soaked in 5 M HCl, stirred for 6 h at room temperature, filtered with a vacuum system, and rinsed intensively with double-distilled H_2_O until a neutral pH was reached. Then, the powder was dried at 110 °C for 12 h and calcined at 550 °C for 6 h. The purified DE was cooled to room temperature, crushed, ground with a mortar and pestle, and stored until investigation. 

#### 2.2.2. Hydrothermal Treatment of DE 

In a typical synthesis process, 5 g of purified DE was dispersed in 50 mL of a 10 M NaOH aqueous solution. The suspension was stirred for 2 h at room temperature. Then, 1 g of CTAB was added to distilled H_2_O, followed by sonication for 15 min, and the mixture was poured into the DE suspension. The resulting suspension (DE + NaOH + CTAB in H_2_O) was poured into a Teflon-lined autoclave, which was tightly sealed and kept in an oven at 160 °C for different time periods (2–8 h). Then, the reaction mixture was left to cool to room temperature. The liquid portion was subsequently decanted, whereas the solid was mixed with distilled water and filtered with a vacuum pump system. During the filtration, the powder was rinsed thoroughly with distilled H_2_O. The solid part was dried in an oven at 60 °C for 24 h and calcined at 550 °C for 6 h. Finally, the powder was transferred to a glass bottle for characterization. To examine the effect of the surfactant on the formation of DBMs, similar syntheses were conducted with various amounts of surfactant (CTAB, SDBS). 

#### 2.2.3. Preparation of Catalyst/DBM Samples

DBM was used as a support to deposit silver and manganese oxide.

##### Ag/DBM Preparation

In a typical procedure, 200 mg of DBM was dispersed in an AgNO_3_ solution so that the weight ratio of Ag to DBM was 5 wt. %. The mixture was stirred for 1 h. Then, an NaBH_4_ solution was added so that the molar ratio of NaBH_4_ to AgNO_3_ was 2:1. The reaction was stopped after 30 min. The solid was recovered through vacuum filtration, washed intensively with distilled H_2_O, and dried at 60 °C for 12 h. The Ag/DBM catalyst was annealed at 400 °C for 4 h under air conditions with a ramp rate of 1 °C/min. 

##### Mn/DBM Preparation

In a typical procedure, 200 mg of DBM was dispersed in an Mn(NO_3_)_2_·6H_2_O solution so that the weight percentage of Mn to DBM was 10 wt. %. The mixture was stirred for 1 h. Then, the solution was slowly heated to 70 °C in an oil bath and stirred continuously for 12 h. The obtained solid was dried at 110 °C for 12 h. Finally, the dried powder was calcined at 400 °C for 4 h under air conditions with a ramp rate of 1 °C/min. 

#### 2.2.4. Material Characterization

The chemical compositions of the original DE and DBM were measured using an X-ray fluorescence spectrometer (Lab Center XRF-1800, Shimadzu, Da Nang, Viet Nam). The morphology and microstructure of all samples were characterized via secondary electron microscopy (Scanning Electron Microscope, JSM-6010 Plus/LV, JEOL, Da Nang, Viet Nam). The phase and crystallinity of the powders were investigated via X-ray diffraction (XRD) analysis (SmartLab, Rigaku, Da Nang, Viet Nam) using a diffractometer with CuKα radiation (λ = 1.54178 Å) at 40 kV and 40 mA and with 2θ scanning from 5° to 70°. The specific surface area and pore volume of the powders were determined via N_2_ adsorption–desorption analysis using an ASAP 2020 (Micromeritics, Da Nang, Viet Nam) apparatus. Prior to the analysis, the samples were degassed at 150 °C for 4 h under vacuum conditions. The specific surface area was calculated by BET (Brunauer-Emmett-Teller) equation in the range of relative pressure (P/P_o_) from 0.05 to 0.35. The pore volume and pore size distribution was calculated by using Barrett-Joyner-Halenda (BJH) method from the desorption isotherm data.

#### 2.2.5. Catalytic Activity Test 

The as-prepared DBM (synthesized via hydrothermal for 8 h) was used as a support to deposit silver and manganese oxide. Catalyst/DBM was subjected to a catalytic test for the toluene total oxidation reaction. A fixed-bed stainless-steel tubular reactor (BTRS reactor, Parker Autoclave Engineers) was used under the atmospheric pressure. The temperature of the reactor was continuously monitored by two thermocouples placed inside and outside the reactor. A saturated mixture of gas containing toluene vapor was generated by bubbling 10 mL/min of a N_2_ gas stream through a toluene saturator at 5 °C. This toluene-containing N_2_ stream was mixed homogeneously with a clean dry airflow. The total flow rate of the feed (air + N_2_ + toluene) was 105 mL/min, and the concentration of toluene in the airstream was 400 ppm. One-hundred milligrams of the catalyst/DBM was filled into the stainless-steel tube with a gas hourly space velocity (GHSV) of 40000 h^−1^. Prior to the tests, the catalysts were pretreated in an airflow of 10 mL/min at 250 °C for 2 h. The reaction was first conducted at 30 °C for 1 h to stabilize the system. Then, the temperature was increased gradually, with a ramp rate of 1 °C/min, from room temperature to 400 °C. The reaction products were analyzed online using a gas chromatography system (Agilent 7890B) equipped with FID and TCD detectors. The principal products of the catalytic oxidation of toluene over the Mn/DBM and Ag/DBM catalysts were carbon dioxide and water. No other product was observed. The carbon balance was nearly 100 ± 5%. The toluene conversion to CO_2_ (*η*) was calculated using the following formula:

(1) $$\eta =\frac{{\left[CO_{2}\right]}_{out}}{7\times {\left[toluene\right]}_{in}}\times 100,$$

where [*toluene*]*_in_* and [*CO*_2_]*_out_* represent the concentrations of toluene in the inlet gas stream and CO_2_ in the outlet gas stream, respectively.

## 3. Results and Discussion

### 3.1. Structure and Formation Mechanism of DE WBL Microspheres

**Figure 1:**
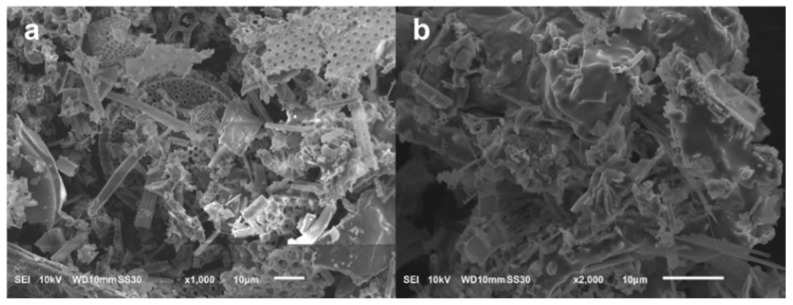
SEM images of (**a**) raw diatomite (DE) and (**b**) DE immediately before the hydrothermal treatment.

The morphologies of raw DE and pre-hydrothermal treatment samples are shown in Figure 1. The raw DE exhibits the common morphologies of diatoms (Figure 1a) [2]. Among the various shapes, the rod-like and disk-like shapes were the most abundant. The disk-shaped constituents contained many sub-micron holes, which is a recognized characteristic of diatoms. The size of the diatomic constituents was not homogeneous, varying from a few micrometers to >10 µm. The original shape of the DE was modified after stirring for 2 h in a 10 M NaOH solution at room temperature (Figure 1b). DE particles were partially deformed, merged with each other, and formed a bulky block. This phenomenon resulted from the dissolution of DE in the NaOH solution.

**Figure 2:**
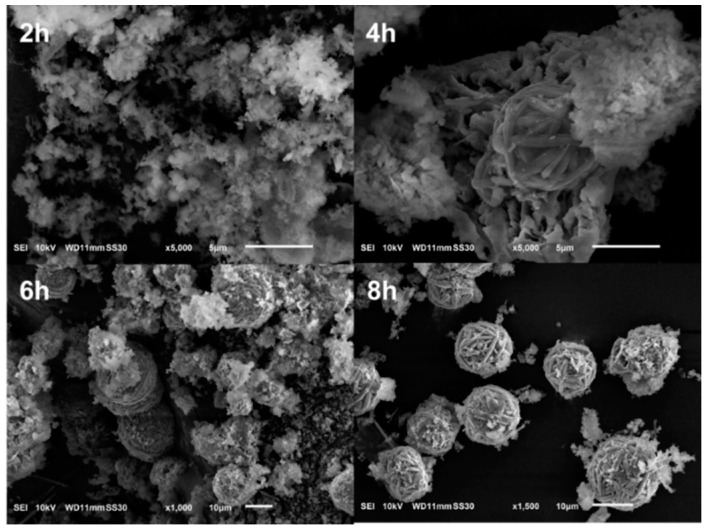
SEM images of diatomite-based material (DBM) after hydrothermal treatment for 2, 4, 6, and 8 h.

After the hydrothermal treatment, the original shape of the DE had completely disappeared, and new morphologies had emerged (Figure 2). After 2 h of treatment, a sheet-like mesophase had appeared. After 8 h of treatment, the mesophase had gradually transformed into WBL microspheres, with diameters ranging from 7 to 10 µm. It appears that these spheres were formed by the aggregation of rod-like fibers. Interestingly, nearly monodispersed spheres were obtained, which has been the main objective of many studies [28]. The SEM images illustrate the gradual disappearance of the mesophase. After 8 h, only a small amount of residual mesophase was observed in the vicinity of the microsphere surface. 

The main chemical components of the raw DE and DBM synthesized after hydrothermal treatment for 8 h are presented in Table 1. The data agree with the literature [29,30], indicating that Si was the dominant element. Other elements were also present, in small quantities. After the hydrothermal treatment, the proportion of components had changed. The amount of Si had significantly decreased, while the amounts of Fe, Ca, and Al had increased. The Na content in DBM was 4.17 wt. %, which resulted from the incorporation of Na from the solution into the precipitate.

**Figure 3:**
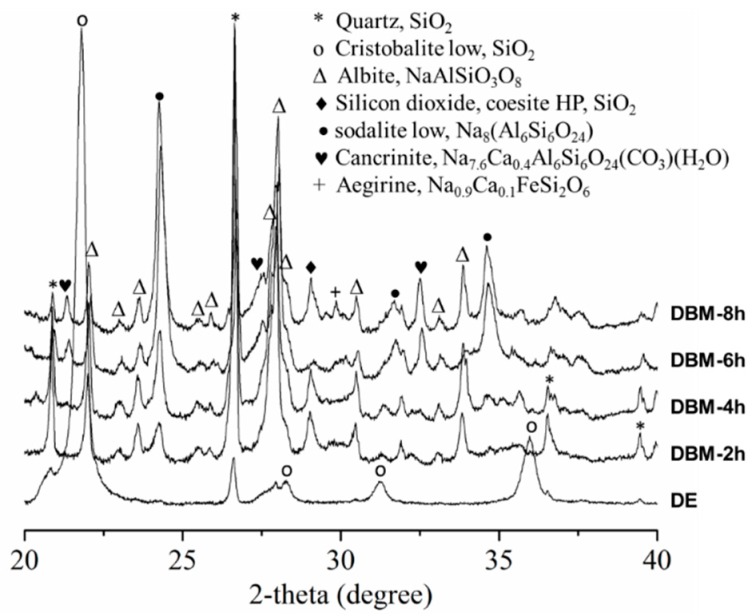
X-ray diffraction (XRD) diffractograms of DE and DBM after hydrothermal treatment for 2, 4, 6, and 8 h.

XRD patterns of the raw DE and DBM samples are presented in Figure 3. For the raw DE, the main signals were from quartz (JCPDS card no. 033-1161), low-cristobalite (JCPDS card no. 076-0941), and albite (NaAlSiO_3_O_8_, JCPDS card no. 089-6426) [31]. The XRD patterns of DBM exhibit many diffraction peaks, indicating the evolution and complexity of the crystalline phases. The significant reduction of the peak intensity of low-cristobalite after 2 h may have been due to the dissolution of SiO_2_ in the strong basic medium. Concurrently, the peak intensity of quartz and albite increased significantly. New crystalline elements were identified, such as silicon dioxide (coesite HP, SiO_2_, JCPDS-083-1832), low-sodalite (Na_8_(Al_6_Si_6_O_24_), JCPDS-071-5356), aegirine (Na_0.9_Ca_0.1_)Fe(Si_2_O_6_), JCPDS-076-2562), and analcime-C (Na(Si_2_Al)O_6_·H_2_O, JCPDS-041-1478). Among these crystal elements, three were made up of Na, Ca, Fe, and Al ions incorporated into the crystalline structure. The Na mainly originated from the NaOH used in the hydrothermal treatment, and the Al, Fe, and Ca were from the original DE source. When the hydrothermal treatment time increased to 6 h, the DBMs evolved, with the appearance of cancrinite (Na_7.6_Ca_0.4_Al_6_Si_6_O_24_(CO_3_)(H_2_O), JPCDS-073-0540) peaks at 2θ = 21.3°, 27.6°, and 32.5°, and increases in the intensities of the low-sodalite peaks at 31.7° and 34.7°.

After 8 h of treatment, the silicate species were rearranged and new mineral crystals were generated. During this process, Na^+^, Ca^2+^, Fe^2+^, and Al^3+^ ions were inserted into the new structures. The insertion may have been due to the binding between positive metal ions and negative silicate species in the solution. Similar incorporation phenomena have been previously reported [31,32]. 

#### 3.1.1. Formation Mechanism of WBL Microspheres

**Figure 4:**
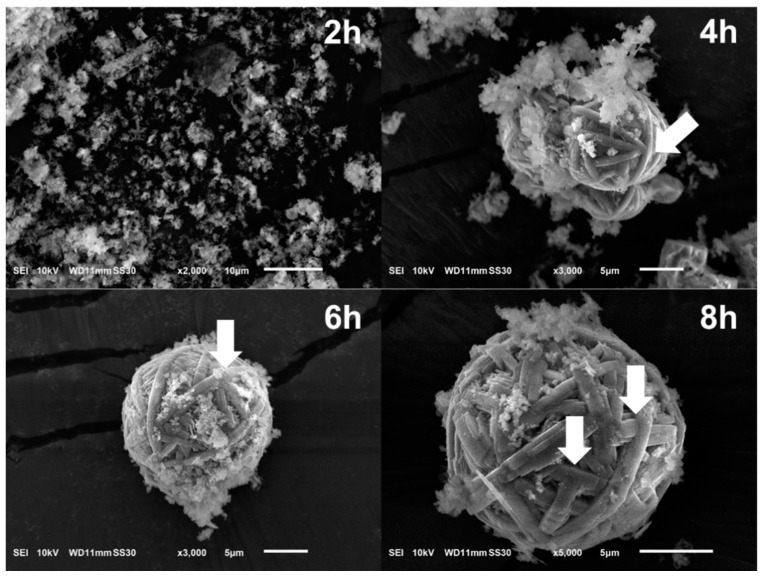
DBM obtained after hydrothermal treatment for various durations. The white arrows indicate the positions where the fibers branched.

The hydrothermal treatment modified the original morphology of DE and formed new structures [17]. The microspheres appeared after 3–4 h of treatment and comprised many fibers with dimensions of 0.25 × 15 µm^2^ (Figure 4). The fibers appeared to develop from the mesophase clusters in two ways: (1) simultaneous crystallization in the form of sub-micron fibers and (2) branching, i.e., one fiber grown from another. Together, the fibers ultimately formed a WBL microsphere. 

To explain the formation mechanisms of crystalline materials under hydrothermal treatment, two popular mechanisms have been proposed: dissolution–recrystallization and pseudomorphic transformation [28,33]. The latter process occurs when the initial shape of the material is conserved while new structures are created layer-by-layer. This phenomenon was not observed in the present study. Thus, the formation of microspheres was considered to follow a dissolution–recrystallization process [34,35]. 

**Figure 5:**
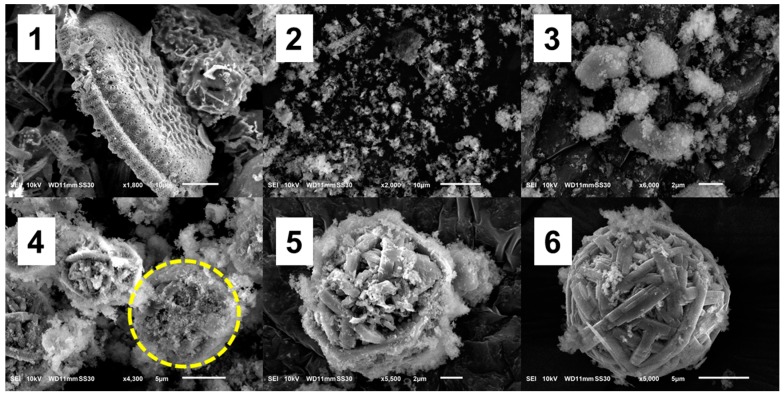
Formation of WBL microspheres from raw DE (**1**), mesophase (**2**), mesophase cluster (**3**), beginning of crystallization (**4**), fiber formation (**5**), and the WBL microsphere (**6**). The yellow circle shows a microsphere with a crystalline interior and a mesophase at the boundary.

Upon hydrothermal treatment, the dissolution of DE led to a high concentration of Si-based species, which aggregated and formed new solid states. The formation mechanism involves the following steps, as shown in Figure 5.

1. DE dissolves under the attack of hydroxide ions.
2. When the concentration of silicate species reaches supersaturation under the hydrothermal condition, mesophase structures (sheet-like) of silicates are formed and coalesced into clusters.
3. Crystallization begins at high temperature and pressure under hydrothermal conditions. With the preferential binding of CTA^+^, these mesophases favorably crystallize into microfibers. Then, these fibers curve to minimize the free energy and eventually form a WBL microsphere.

**Figure 6:**
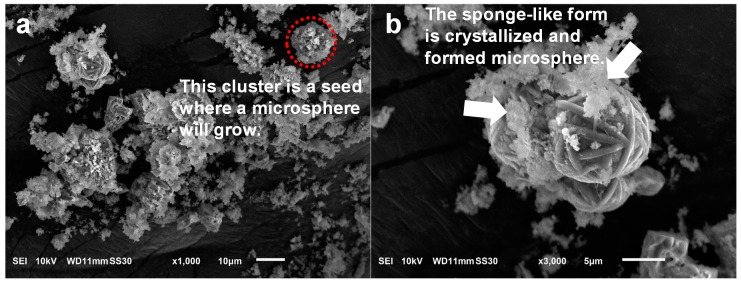
SEM images of DBM after hydrothermal treatment of DE for 4 h. (**a**) Microsphere growth from a cluster (dotted red circle); (**b**) mesophase on the surfaces of the microspheres.

Additional evidence supporting the dissolution and regrowth mechanism is the presence of microspheres that were crystalline in the interior while the boundary was not completely crystallized (Figure 5(4) and Figure 6a). This phenomenon can be explained by the following steps, which are similar to those presented previously. First, the sheet-like mesophase coalesced and formed spherical clusters. Then, crystallization began inside the clusters. As fibers elongated, they curved and formed WBL microspheres. The dissolution–recrystallization mechanism appears to be reasonably established in the present conditions. DE, which is mainly composed of amorphous SiO_2_, is easily attacked by hydroxide ions, which originate primarily from NaOH and partly from H_2_O dissociation [36]. The surface of the DE first dissolved; then, the entire particles gradually dissolved into the solution. The dissolution of amorphous SiO_2_ in highly alkaline NaOH involves two steps [37,38]: the surface SiO_2_ dissolves in H_2_O and then forms Si-OH according to the following reaction:

Si–O–Si + H_2_O → 2Si–OH.

Then, deprotonation occurs in NaOH:

Si–O–Si + H_2_O + 2OH^−^ → 2Si-O^−^ + 2H_2_O.

The dissolution of silica is endothermic. Thus, a higher temperature leads to a higher dissolution rate [38]. Additionally, the dissolution rate is proportional to the density of Si–O^−^ on the surface of SiO_2_ structures [37]. Therefore, with a higher concentration of NaOH, the dissolution proceeds more rapidly. Finally, the solution of amorphous SiO_2_ should contain different types of species, such as orthosilicic acid, pyrosilicic acid, metasilicic acid, disilicic acid, and other polymeric species [39]. Through this mechanism, DE dissolved and formed different silicate species [40]. The monomer species dominated at the beginning of the dissolution process, and then dimers and polymeric species were gradually formed. The latter species condensed and precipitated, generating clusters and eventually particles (amorphous and crystalline).

As CTAB was part of the reaction mixture, it also had important effects on the crystallization and morphology of the DBM. The CTAB molecules dissociated into CTA^+^ ions and were adsorbed on the surface of growing crystals, promoting the crystal growth in a preferential direction. This preferential adsorption phenomenon has been previously observed during the formation of ZnO nanorods [41] and PbWO_4_ crystal synthesis [42]. The detailed role of CTAB in DBM formation is discussed below.

#### 3.1.2. Effects of Cetyltrimethylammonium Bromide on DE-Based Materials Formation

To understand the formation of WBL microspheres, it is necessary to understand the interaction between CTAB and silicate species in the reaction mixture. Until now, no report has discussed this interaction under similar experimental conditions. In this study, the hydrothermal treatment was conducted at 160 °C for several hours with 10 M NaOH. CTAB was used at 10^−2^ M to ensure the presence of micelles in the aqueous solution [43]. The hydrothermal conditions may have affected the assembly nature of CTAB in the reaction mixture. CTAB may have been present as an organized structure or as free molecules. Kreske reported that surfactants remained intact after hydrothermal treatment at 210 °C for up to 48 h [13]. In agreement with Kreskes’s report, we observed that during the filtration step to recover solid DBM, the filtrate contained foams, indicating the presence of intact CTAB molecules after the hydrothermal treatment. If CTAB existed as hexagonal arrays, the DBM would have ordered hexagonal pores, as reported by Kreske [13]. The hexagonal pore structure should create DBM with a large specific surface area. Hexagonal structures only exist at a high concentration of CTAB, which was not the case under the present conditions. Additionally, auto-assembled structures of CTAB are only formed at high concentrations [44,45]. Monnier reported that the concentration of CTAB must be 25–70 wt. % to favor the formation of hexagonal structures in an aqueous solution [17]. Similarly, the lamellar phase of CTAB only exists at very high concentrations, i.e., ≥70 wt. %. Thus, it is logical to suggest that under the present conditions, the CTAB existed as free, positively charged ions (CTA^+^).

DE is mainly composed of SiO_2_, which easily dissolves in a strong alkaline solution. The dissolution generates various negatively charged silicate species, including monomeric (Si(OH)_2_O_2_^2−^, Si(OH_3_)O), dimeric (Si_2_O_7_^6−^), trimeric (Si_3_O_7_^2−^), and tetrameric species [45]. These species condense and form polymeric rings and multidentate polyanionic clusters [46]. The pK_a_ of monosilicic acid is approximately 9.5 [45]; thus, silicate species are likely deprotonated and negatively charged in a strongly alkaline solution. In particular, polymeric silicate species can function as multidentate binding substrates for CTA^+^ ions. CTAB is a cationic surfactant that can bind to negatively charged silicate species in solution or at the surface of crystals by binding to SiO^−^ [47]. The binding occurs through electrostatic interactions and should be geometrically preferential to control the growth direction of the crystal. CTA^+^ ions horizontally bind to the multivalent negatively charged polyanions formed by the silicate species [17], facilitating growth along the fiber and restricting growth in the wider direction. This binding mode of CTA^+^ has been reported for the synthesis of Au nanorods [48] and hydroxyapatite [49].

Additionally, the tetrahedral geometry of the silicate species unit promotes the interaction with CTA^+^ ions owing to geometric matching [50]. Yan et al. suggested that the binding of CTA^+^ ions to phosphate moieties is facilitated by geometric matching [51]. A similar concept is commonly used in drug-receptor theory. Thus, CTA^+^ ions favorably bound to silicate species via stereochemistry and electrostatic interactions. These bindings occurred during the precipitation process and generated microfibers. When these fibers became long enough, they curved and eventually formed WBL spheres. The formation of spheres with a WBL morphology is not commonly reported in the literature. Recently, Xiuxiu reported the formation of WBL Au nanoparticles [52]. However, templating compounds were not used, and the formation mechanism involved aurophilic interactions among Au^I^ complexes. In contrast, in the present study, CTAB molecules played a crucial role in forming WBL microspheres. 

#### 3.1.3. Effects of CTAB Concentration on Morphology and Crystal Structure

**Figure 7:**
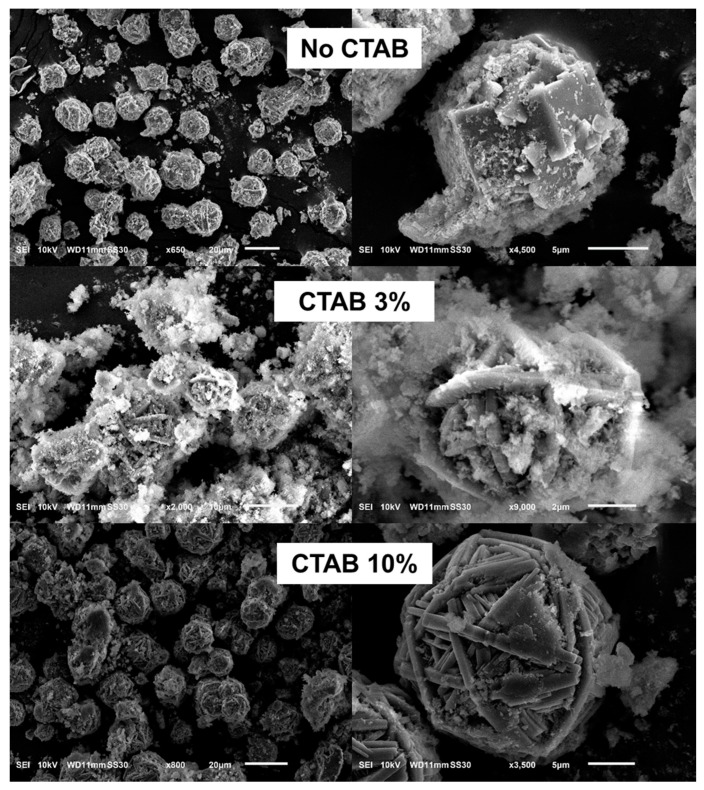
SEM images of hydrothermally treated DE: no CTAB (**top row**); 3 wt. % CTAB (**middle row**); 10 wt. % CTAB (**bottom row**).

SEM images of DBM synthesized in the absence and presence of CTAB are shown in Figure 7. Without CTAB (top row), the DBM was well crystallized into a rectangular crystal of approximately 5 µm. In contrast, the presence of CTAB elongated the crystals, leading to the formation of fibers that folded and formed wool-ball like microspheres. When the CTAB concentration increased, less mesophase was present in the proximity of the microsphere surface (bottom row in Figure 7). The moderate concentration of CTAB indicates that a large amount of mesophase clusters were adsorbed on the fibers. Thus, a sufficient amount of surfactant was needed to form WBL microspheres and accelerate the crystallization. As mentioned previously, the CTAB directed the growth of the fibers. Surprisingly, the quantity of CTAB did not affect the crystal structure of the DBM, as shown in Figure S1. It is considered that the quantity of CTAB was not high enough to generate an obvious difference. 

#### 3.1.4. Effect of Surfactant Type (Cationic Versus Anionic)

**Figure 8:**
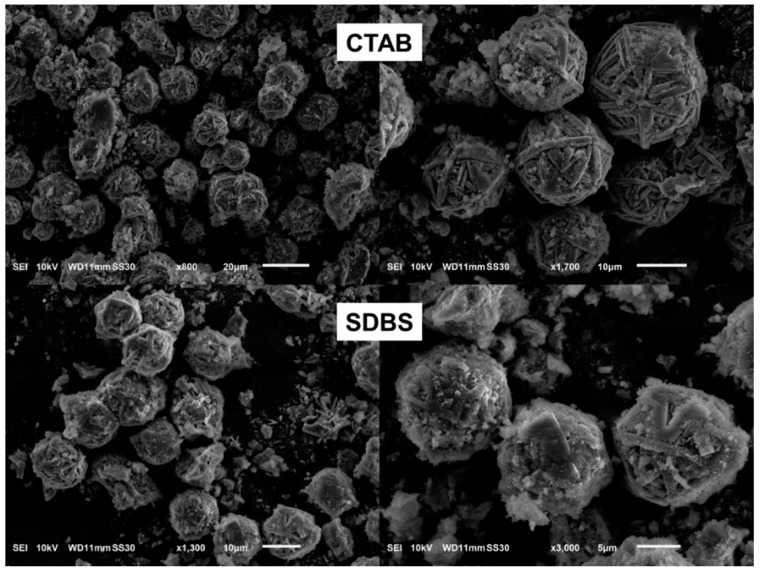
SEM images of DBM after hydrothermal treatment in the presence of 10 wt. % CTAB (**top row**) and 10 wt. % SDBS (**bottom row**).

To evaluate the effects of a surfactant charge on the formation of microspheres, SDBS was used. SBDS is negatively charged, whereas CTAB is positively charged. As indicated by the SEM images in Figure 8, the CTAB was more effective than the SDBS for controlling the shapes of the elongated fibers and the wool-ball like microspheres. In the presence of CTAB, the microspheres were formed with neater fibers. In the case of SDBS, there were more mesophase clusters surrounding the fibers, while rectangular crystals were still observed. The reduced amount of the mesophase indicates that the binding of CTAB on silicate species was more effective than the binding of SBDS for controlling the morphology and facilitating the crystallization. 

This is understandable considering that the interactions between CTA^+^ and silicate species are stronger than those between SDBS and silicate species. Owing to its negative charge, SDBS does not bind to silicate via electrostatic forces. Instead, SDBS probably interacts weakly with silicate through van der Waals interactions and H bonds. H-bonds may form between silanol groups of silicate and the headgroup of SDBS. The combination of these interactions keeps SDBS on the surface of silicate species and directs the growth of the crystal fibers. A similar effect of SDBS on the crystal size has been observed in PbWO_4_ synthesis [42].

However, the charge of the surfactants used in this study did not have any observable effect on the crystal structure (Figure S2, supplementary). It appears that the crystal structure was determined by the reaction conditions rather than by the presence of surfactants. 

#### 3.1.5. Textural Properties

The hydrothermal treatment not only modified the crystal structure and morphology of the DBM but also its textural properties. Table 2 presents the specific surface area and total pore volume of the samples. The N_2_ adsorption measurements indicated that the hydrothermal treatment significantly changed the porosity of the original DE, making it more porous. The specific surface area of the DE was very small (3 m^2^/g), and the total pore volume was almost 0 cm^3^/g, whereas those of the DBM were significantly larger. In particular, the specific surface area of the DBM was 25–60 times larger than that of the DE, depending on the treatment duration. Interestingly, the DBM after 2 h had the largest specific surface area (185 m^2^/g) and total pore volume (0.32 cm^3^/g). When the treatment time increased to 8 h, the surface area and pore volume decreased to 84 m^2^/g and 0.18 cm^3^/g, respectively. This reduction may have been due to crystallization.

**Figure 9:**
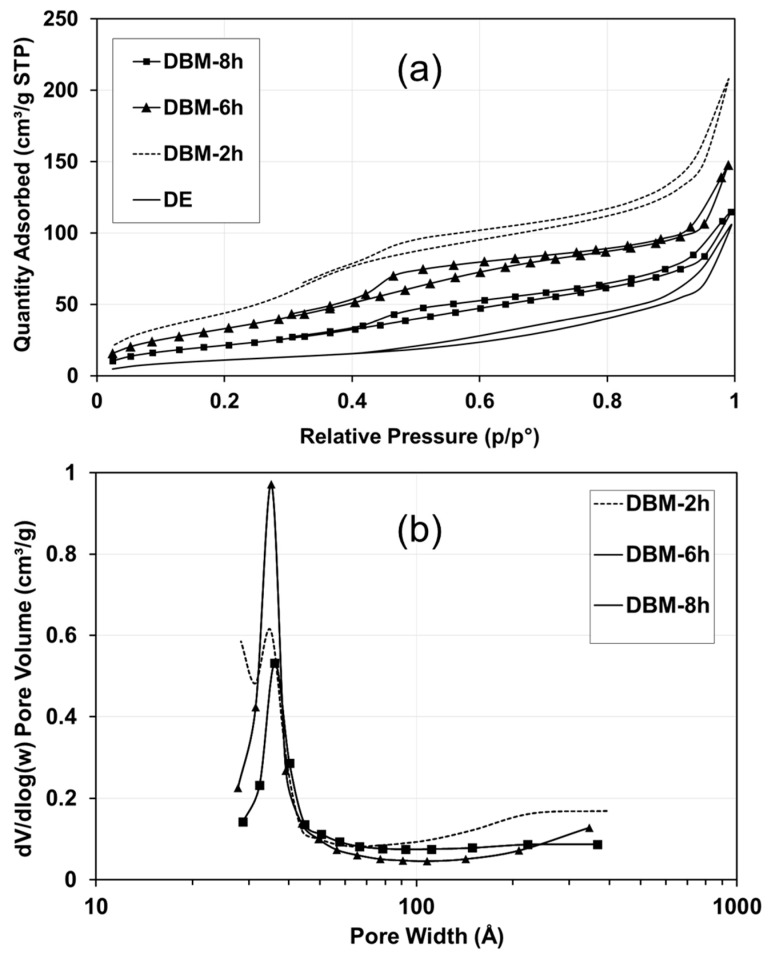
Porosity of DE and DBM after hydrothermal treatment for 2, 6, and 8 h: (**a**) N_2_ adsorption–desorption isotherms and (**b**) pore volume with respect to the pore width calculated according to BJH method using the data from the desorption branch.

Figure 9a shows the N_2_ adsorption–desorption isotherms of the DE and DBM. The isotherms of all the DBMs exhibited hysteresis loops. According to the Brunauer classification [53], the loops are due to the type IV isotherm with H4-type hysteresis. Figure 9b shows the pore volume with respect to the pore size. The DBM after hydrothermal treatment for 2, 6, and 8 h was mainly characterized by mesopore diameters centered at 3.5–4.5 nm. Thus, the mesoporous properties of the DBMs appear to be suitable for application as a catalyst support for the gas-phase heterogeneous reaction. 

### 3.2. DE WBL Microspheres as Support for Metallic Catalyst 

#### 3.2.1. Crystalline Phase of Ag/DBM and Mn/DBM Catalysts

**Figure 10:**
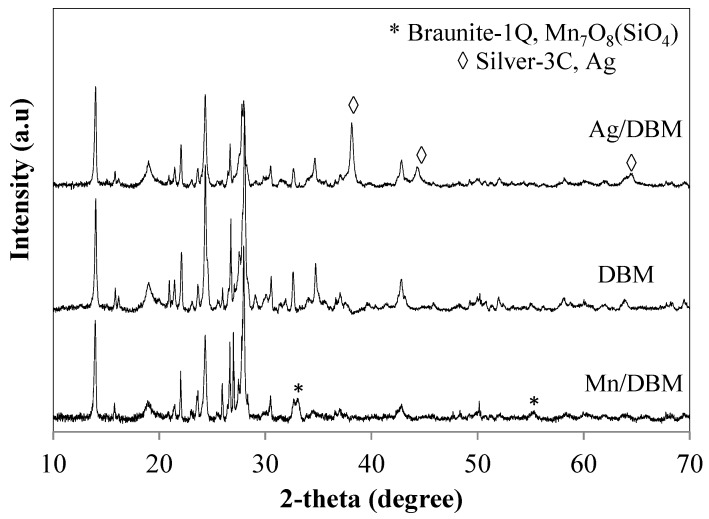
Diffractograms of DBM, Ag/DBM, and Mn/DBM catalysts.

After deposing manganese on DBM, the crystalline phase of manganese hexamanganese (III) octaoxide silicate (braunite-1Q, Mn_7_O_8_(SiO_4_) jcpds no 089-5664) was identified at 2θ of 33° and 55° (Figure 10). The diffraction peak of SiO_2_ coesite HP at 29° disappeared. The formation of braunite may have resulted from the interaction between manganese oxide and SiO_2_ during the calcination process at 400 °C. For the Ag/DBM catalyst, metallic Ag was detected at 2θ = 38.1°, 44.3°, and 64.4°.

#### 3.2.2. Catalytic Performance of Ag/DBM and Mn/DBM Catalysts

**Figure 11:**
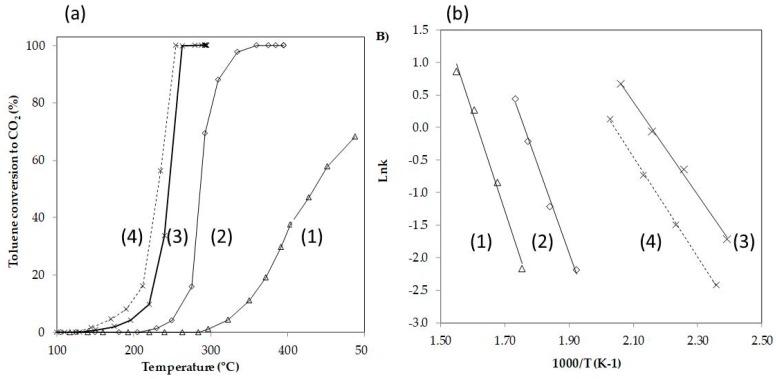
(**a**) Catalytic performance and (**b**) Arrhenius plots of the DBM-based catalysts ((1) DBM[toluene]_in_ = 400 ppm, (2) Ag/DBM[toluene]_in_ = 400 ppm, (3) Mn/DBM [toluene]_in_ = 400 ppm, and (4) Mn/DBM [toluene]_in_ = 130 ppm).

The efficiencies of Ag/DBM, Mn/DBM, and DBM to catalyze the combustion of toluene are presented in Figure 11. As shown, the DBM was slightly active at a high temperature of 300 °C. The toluene conversion into CO_2_ barely reached 70% at a high temperature of 500 °C. Additionally, the results indicated that the Ag/DBM and Mn/DBM catalysts were both active at lower temperatures. The toluene was completely converted into CO_2_ at 280 °C for Mn/DBM and at 350 °C for Ag/DBM. Accordingly, the Mn/DBM catalyst exhibited high activity for the total oxidation of toluene. For lower toluene inlet concentration, the complete oxidation temperature could reduce to 255 °C. Table 3 presents the catalytic performance of the Ag/DBM and Mn/DBM catalysts for the oxidation of toluene. They are compared with previously reported catalysts. The data indicate that the DBM alone was not very active in the toluene oxidation reaction and that Mn/DBM and Ag/DBM were significantly more active. The T90 values in Table 3 (referring to the temperature at which the toluene conversion was 90%) varied by approximately 200–300 °C depending on the catalysts, toluene concentration (ranging from 200 to 1000 ppm), and space velocity (ranging from 20,000 to 80,000 h^-1^). The T90 value obtained in this study indicates that the Mn/DBM catalyst is highly active compared with other catalytic systems. For further comparison of the catalytic activity of the catalysts, the values of energy activation (Ea) of the catalysts were determined by Arrhenius equation according to the previous report [54,55,56], supposing that the toluene oxidation followed the first-order reaction toward toluene concentration and zero-order toward oxygen concentration because of the high molar ratio of oxygen to toluene (>400 times). The linear plots were obtained for all the catalysts (Figure 11B). The Ea values of the DBM, Ag/DBM, and Mn/DBM catalyst were 125, 115, and 66 KJ/mol, respectively. Here, the best Mn/DBM catalyst for toluene oxidation shows the lowest Ea value. Thus, the results suggest that DBM can be used as an efficient catalyst support. However, a detailed investigation of the characteristics of the DBM is essential before any definite conclusion can be drawn. Some properties should be considered, including the rehydration during catalyst applications (because H_2_O is generated inside the reactor), as well as the chemical and thermal stability of the supports. 

## 4. Conclusions

For the first time, the synthesis of WBL microspheres via a hydrothermal method was demonstrated. The transformation of DE during a hydrothermal treatment process was systematically investigated. First, the DE was dissolved to form a mesophase and clusters. Under the effects of the temperature, pressure, and surfactant, the mesophase clusters crystallized into WBL microspheres. These microspheres comprised an assembly of fibers. The presence of the surfactant was a prerequisite for generating WBL microspheres. The cationic surfactant facilitated crystallization, whereas an anionic surfactant was less effective. Without a surfactant, microspheres were only generated from clusters of rectangular crystals, not from fiber crystals. Such WBL spheres have a significantly larger specific surface area than the original DE; thus, they can be used as effective metallic catalyst supports for the total oxidation reaction of toluene.

## Figures and Tables

**Table 1:** Principal components (in weight percentage) of raw diatomite (DE) and DE-based materials (DBM) obtained after hydrothermal treatment for 8 h after annealing at 550 °C (HT-8h).

Components	DE Raw (%)	DBM (HT-8h) (%)
Si	87.50	47.51
Fe	4.94	19.73
Ca	1.98	9.80
Al	2.30	11.66
K	2.71	3.81
P	0.54	2.38
Ti	-	0.90
Na	-	4.17

**Table 2:** Specific surface area and total pore volume of the DE and DBM.

Sample	Specific Surface Area (m^2^/g)	Total Pore Volume (cm^3^/g)
DE	3.1	0.001
DBM-2h	185.1	0.32
DBM-6h	133.5	0.23
DBM-8h	84.0	0.18

**Table 3:** Catalytic performance of different catalysts for the oxidation of toluene.

Catalyst	Specific Surface Area (m^2^/g)	Toluene Concentration (ppm)	GHSV ^1^	T90 ^2^ (°C)	Ref.
Mn/DBM Ag/DBM	75 64	160 400 400	40,000 h^−1^ (or 60,000 cm^3^⋅g^−1^⋅h^−1^)	250 260 315	This study
Mn/DE	33	1000	30,000 cm^3^⋅g^−1^⋅h^−1^	279	[5]
MnO_x_/Al_2_O_3_ CuO–MnO_x_/Al_2_O_3_ CeO_2_–MnO_x_/Al_2_O_3_	53 50 79	1000	200,000 cm^3^⋅g^−1^⋅h^−1^	>300 270	[49]
Mn_3_O_4_ Mn_2_O_3_ MnO_2_ 0.5 wt. % K/Mn_3_O_4_	18 7 3 16	1000	15,000 cm^3^⋅g^−1^⋅h^−1^	270 280 340 250	[50]
KMn/SBA-15	500–600	1600	-	300	[51]
5% MnO_x_/TiO_2_ MnCuO_x_/TiO_2_	50	500	5000 h^−1^	230	[57]
15 wt. % Cu/Al_2_O_3_ 5 wt. % Cu/Al_2_O_3_	182 203	1000 160	4800 cm^3^⋅g^−1^⋅h^−1^	290 260	[58]
FeMn oxide MnO_x_	178 14	500	50,000 h^−1^	197 253	[59]

^1^ GHSV: gas hourly space velocity; ^2^ T90: temperature at which 90% of toluene was converted.

## Supplementary Materials

The following are available online at https://www.mdpi.com/2079-4991/10/2/339/s1, Figure S1: XRD Diffractograms of diatomite-based material after a hydrothermal treatment of 8 h in the presence of different amounts of CTAB 0 wt. %, 3 wt. %, and 10 wt. %., Figure S2: XRD Diffractogram of diatomite-based material after a hydrothermal treatment of 8 h at 160 °C in the presence of CTAB or SDBS.
