# Supplementary material for: Synthesis of Diatomite-Based Mesoporous Wool-Ball-Like Microspheres and Their Application for Toluene Total Oxidation Reaction
Source: Nanomaterials (Basel). 2020 Feb 17;10(2):339. doi: 10.3390/nano10020339 (PMC7075114; doi:10.3390/nano10020339)
Supplement: Supplementary file 1 [file nanomaterials-10-00339-s001.pdf]

Supplementary information

Synthesis of Diatomite-Based Mesoporous Wool-Ball-Like Microspheres and their Application for Toluene Total Oxidation Reaction

Quoc-Chon Le [1], Chinh Chien Nguyen [2], Thi Thanh Nhi Le [2], Thierry Lefèvre [3], Minh Tuan Nguyen Dinh [4,*], Sung Hyun Hong [5], Soo Young Kim [5,*] and Quyet Van Le [2,*]

[1] Natural Sciences Department, Duy Tan University, Danang 550000, Vietnam; lequocchon@gmail.com

[2] Institute of Research and Development, Duy Tan University, Danang 550000, Vietnam; nguyenchinhchien@duytan.edu.vn (C.C.N); lethithanhnhi.kh@gmail.com (T.T.N.L.)

[3] Département de chimie, Proteo, Cerma, CQMF, Université Laval, Québec, QC G1V 0A6, Canada; thierry.lefevre@chm.ulaval.ca

[4] Faculty of Chemical Engineering, University of Science and Technology, The University of Da Nang, 54 Nguyen Luong Bang, Da Nang 550000, Vietnam

[5] Department of Materials Science and Engineering, Korea University, 145 Anam-ro, Seongbuk-gu, Seoul 02841, Korea; qhrdjakstp@naver.com

* Correspondence: ndmtuan@dut.udn.vn (M.T. N.D..); levanquyet@dtu.edu.vn (Q.V.L.); sooyoungkim@korea.ac.kr (S.Y.K.)

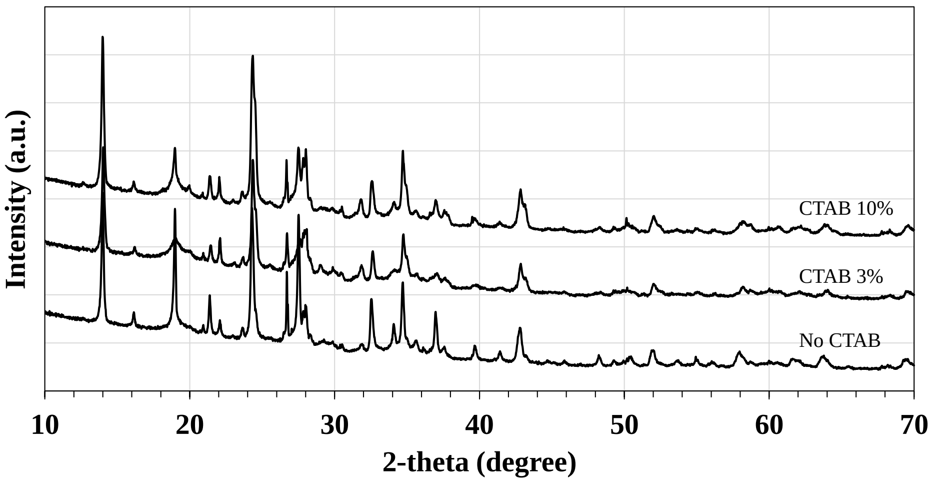

Figure S1. Diffractograms of diatomite-based material after a hydrothermal treatment of 8 h in the presence of different amounts of CTAB 0 wt. %, 3 wt. %, and 10 wt. %.

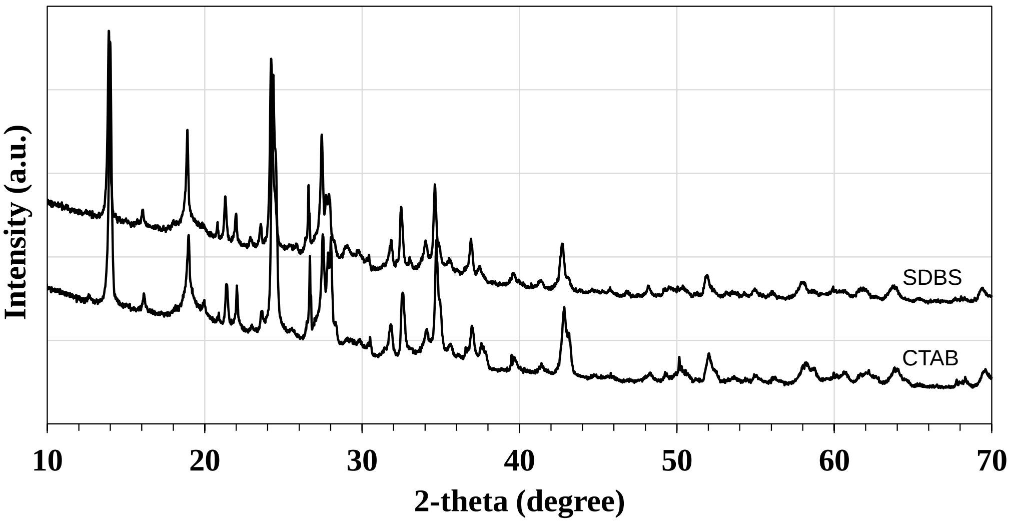

Figure S2. Diffractogram of diatomite-based material after a hydrothermal treatment of 8 h at 160 °C in the presence of CTAB or SDBS
